# TLR9-mediated dendritic cell activation uncovers mammalian ganglioside species with specific ceramide backbones that activate invariant natural killer T cells

**DOI:** 10.1371/journal.pbio.3000169

**Published:** 2019-03-01

**Authors:** Christophe Paget, Shenglou Deng, Daphnée Soulard, David A. Priestman, Silvia Speca, Johanna von Gerichten, Anneliese O. Speak, Ashish Saroha, Yael Pewzner-Jung, Anthony H. Futerman, Thierry Mallevaey, Christelle Faveeuw, Xiaobo Gu, Frances M. Platt, Roger Sandhoff, François Trottein

**Affiliations:** 1 Univ. Lille, Centre d'Infection et d'Immunité de Lille, Lille, France; 2 Centre National de la Recherche Scientifique, Lille, France; 3 Institut National de la Santé et de la Recherche Médicale, Lille, France; 4 Centre Hospitalier Universitaire de Lille, Lille, France; 5 Institut Pasteur de Lille, Lille, France; 6 Institut National de la Santé et de la Recherche Médicale, Centre d'Etude des Pathologies Respiratoires (CEPR), Faculté de Médecine, Université de Tours, Tours, France; 7 Department of Chemistry and Biochemistry, Brigham Young University, Provo, Utah, United States of America; 8 S&D Lipopharma LLC, Provo, Utah, United States of America; 9 Department of Pharmacology, University of Oxford, Oxford, United Kingdom; 10 Institut National de la Santé et de la Recherche Médicale, Lille Inflammation Research International Center, Lille, France; 11 Lipid Pathobiochemistry Group German Cancer Research Center, Heidelberg, Germany; 12 Faculty of Biosciences, University of Heidelberg, Germany; 13 Department of Biomolecular Sciences, Weizmann Institute of Science, Rehovot, Israel; 14 Department of Immunology, University of Toronto, Toronto, Ontario, Canada; 15 Institute of Biomaterials & Biomedical Engineering, University of Toronto, Toronto, Ontario, Canada; National Cancer Institute, UNITED STATES

## Abstract

CD1d-restricted invariant natural killer T (*i*NKT) cells represent a heterogeneous population of lipid-reactive T cells that are involved in many immune responses, mediated through T-cell receptor (TCR)–dependent and/or independent activation. Although numerous microbial lipid antigens (Ags) have been identified, several lines of evidence have suggested the existence of relevant Ags of endogenous origin. However, the identification of their precise nature as well as the molecular mechanisms involved in their generation are still highly controversial and ill defined. Here, we identified two mammalian gangliosides—namely monosialoganglioside GM3 and disialoganglioside GD3—as endogenous activators for mouse *i*NKT cells. These glycosphingolipids are found in Toll-like receptor-stimulated dendritic cells (DC) as several species varying in their *N*-acyl fatty chain composition. Interestingly, their ability to activate *i*NKT cells is highly dependent on the ceramide backbone structure. Thus, both synthetic GM3 and GD3 comprising a d18:1-C24:1 ceramide backbone were able to activate *i*NKT cells in a CD1d-dependent manner. GM3 and GD3 are not directly recognized by the *i*NKT TCR and required the Ag presenting cell intracellular machinery to reveal their antigenicity. We propose a new concept in which *i*NKT cells can rapidly respond to pre-existing self-molecules after stress-induced structural changes in CD1d-expressing cells. Moreover, these gangliosides conferred partial protection in the context of bacterial infection. Thus, this report identified new biologically relevant lipid self-Ags for *i*NKT cells.

## Abstract 2

Although the existence of self-antigens for invariant Natural Killer T (iNKT) cells is widely accepted, their precise nature remains a matter of debate. This study shows that two mammalian ganglioside species activate iNKT cells in a CD1d-dependent manner.

## Author summary

Invariant natural killer T (*i*NKT) cells are a population of unconventional T lymphocytes that activate rapidly during inflammation due to their innate-like features. They are unconventional since they do not react to peptidic antigens (Ags) presented by classical major histocompatibility complex (MHC) molecules; instead, they recognize lipid-based Ags in the context of the MHC class I-like molecule CD1d. While numerous Ags of microbial origins have been described, their endogenous Ags are far less understood and remain a matter of strong debate. Here, we report that engagement of an innate receptor on the Ag-presenting cells leads to modulation of their lipid metabolism. This results in an enrichment of particular glycosphingolipid species that differ in both the nonpolar tail and polar head structures. Among those, two species have the potential to activate *i*NKT cells in a CD1d-dependent manner after further intracellular modifications. Based on these data, we propose a concept that *i*NKT cells can rapidly respond to pre-existing self-molecules after stress-induced changes in CD1d-expressing cells. Given the presence of closely related molecules in some pathological conditions such as cancer, it will be interesting to evaluate the biological relevance of these Ags in disease states.

## Introduction

Type I or invariant natural killer T (*i*NKT) cells are a subset of “innate-like” αβ T lymphocytes that rapidly produce large amounts of cytokines and chemokines and orchestrate the ensuing innate and adaptive immune responses during infection, inflammatory disorders, and cancer [1]. *i*NKT cells recognize self and exogenous lipid antigens (Ags) presented by the quasimonomorphic CD1d molecule expressed by Ag-presenting cells [2]. In contrast to the highly diverse T-cell receptor (TCR) repertoire of peptide-specific/major histocompatibility complex (MHC)-restricted conventional αβ T cells, *i*NKT cells express a semiconserved TCR composed of a unique and invariant TCRα chain, using TCR TRAV11 to TRAJ18 rearrangement in mice (TRAV10 to TRAJ18 in humans), paired with a limited array of Vβ chains [1].

Accordingly, the antigenic diversity and specificity of *i*NKT cells was intuitively believed to be limited. Over the last decade, numerous studies have attempted to identify cognate Ags involved in *i*NKT cell thymic selection and/or in their activation during infection and stressful conditions in the periphery (for reviews, [2,3]). To date, several candidates have been proposed to act as self-Ags for *i*NKT cells, including β-linked glycosphingolipids (GSLs) (isoglobotrihexosylceramide [iGb3] [4,5], gangliosides [6,7] and β-glucosylceramide [β-GlcCer] [8]), and phospholipids [9]. However, the physiological activity of these compounds has either been called into question or demonstrated to have weak and/or subset-specific activity. For example, iGb3 activates some mouse and human *i*NKT [4] and has been proposed to be responsible for *i*NKT cell development [5]. However, mice deficient for the enzyme involved in direct iGb3 anabolism (*A3galt2*) present no *i*NKT cell defect [10]. In addition, iGb3 is virtually undetectable in mouse peripheral tissues [11], and the human gene encoding iGb3 synthase appears to be nonfunctional [12]. β-GlcCer was also proposed as a self-Ag for both human and mouse *i*NKT cells [8]. However, it was subsequently reported that this activity was restricted to mammalian α-linked monoglycosylceramides contaminants [13,14]. Initially thought to be absent in mammals, small quantities of α-linked monoglycosylceramides (e.g., α-galactosylceramide [α-GalCer]) that may act as *i*NKT cell self-Ags have been identified [14]. Although the origin of mammalian α-GalCer is currently uncertain, further evidence suggests that some bacterial communities from the gut microbiota, including *Bacteroides fragilis*, can produce this particular hexosylceramide [15,16]. In addition, diet could also provide a source of these Ags, as recently demonstrated by the presence of α-linked monohexosylceramides in bovine milk [17]. Thus, the repertoire of self-lipids for *i*NKT cells lacks consensus and is incompletely characterized.

The possibility that the precise structure of the lipid chain of *i*NKT self-Ags could be important for their reactivity to the TCR has only been partially investigated. Nevertheless, some GSLs with particular ceramide structures have been shown to carry variable antigenic capacity [8,14]. Ceramide metabolism is tightly regulated by multiple enzyme families, including ceramide synthases (de novo synthesis) [18] and ceramidases (degradation) [19]. The impact of inflammation on the regulation of these enzymes is currently unknown, as well as their potential involvement in *i*NKT cell biology.

We and others have demonstrated that Toll-like receptor (TLR) engagement on/in Ag-presenting cells resulted in lipid metabolism perturbations uncovering self-Ag(s) for *i*NKT cells [20,21]. Specifically, we observed that TLR7/9-mediated activation of dendritic cells (DCs) resulted in the accumulation of charged β-linked GSLs able to activate mouse *i*NKT cells in a CD1d-dependent manner [21], but the precise nature of these activating compounds remained unknown.

Here, we highlighted two particular ganglioside species found in TLR-stimulated DCs that act as *i*NKT cell activators. Importantly, reactivity of the *i*NKT cells to gangliosides is highly dependent on the specific structure of the ceramide backbone, particularly the *N*-acyl fatty acid chain length and its geometry. Both synthetic d18:1-C24:1 GM3 and d18:1-C24:1 GD3 gangliosides activate *i*NKT cells in vitro and in vivo in a dose- and CD1d-dependent manner. These synthetic gangliosides are unlikely to engage *i*NKT TCR in their native forms and require unidentified intracellular molecular changes to become antigenic. Thus, we propose a new concept in which *i*NKT cells can rapidly respond to pre-existing self-molecules after stress-induced structural changes in CD1d-expressing cells. Collectively, our data bring to light a class of self-lipid Ags that can activate *i*NKT cells and that may be relevant during inflammatory and/or pathological settings.

## Results

### Self-derived simple sialylated ganglioside(s) activate(s) *i*NKT cells

**Fig 1:**
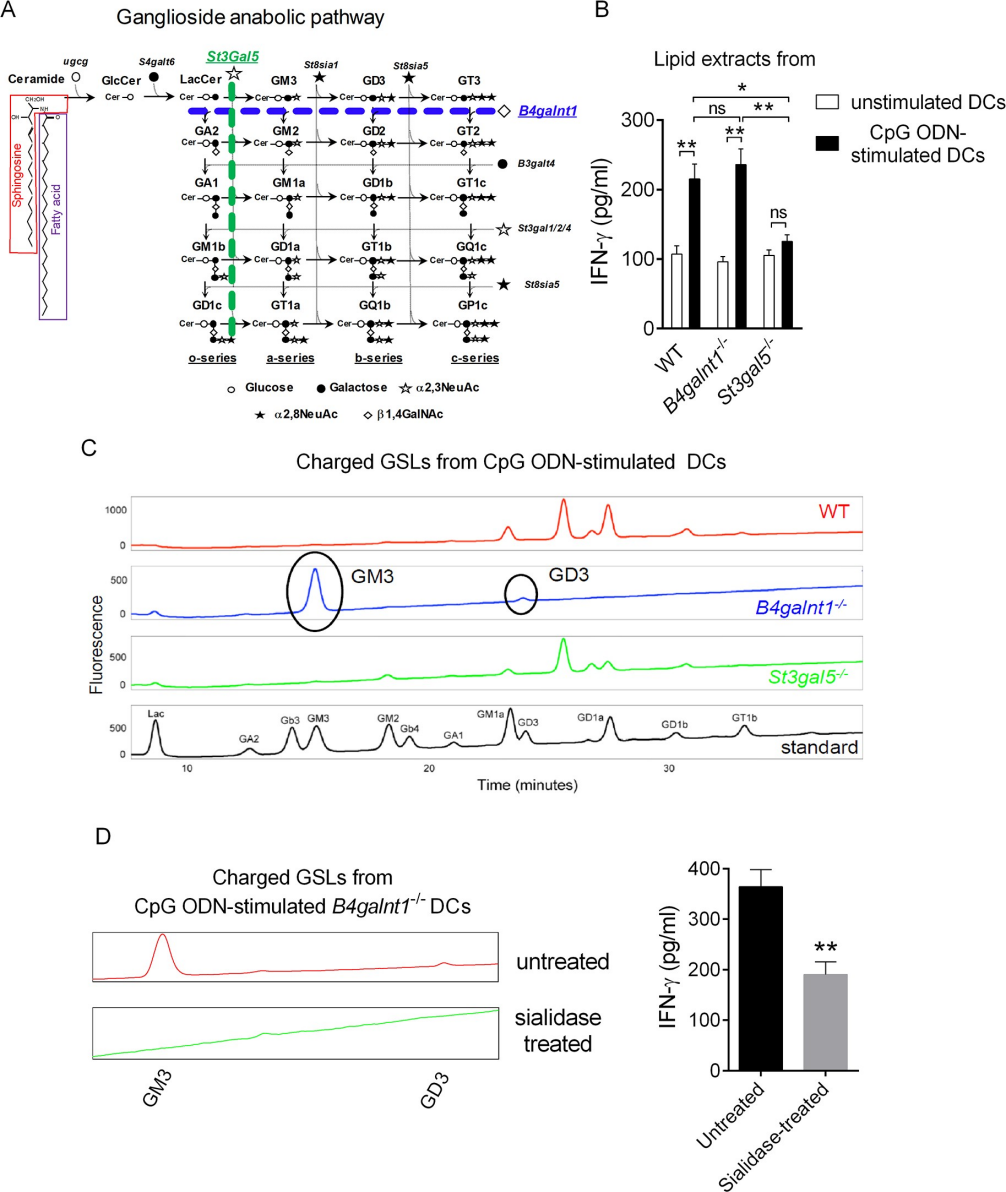
Endogenous ligand(s) for *i*NKT cells in CpG ODN-stimulated DC is/are simple sialylated gangliosides. (A) Ganglioside anabolic pathway including species and key enzymes involved is depicted. (B) Base-treated charged lipids extracted from unstimulated or CpG ODN-stimulated DCs derived from WT, *B4galnt1*^−/−^, or *St3gal5*^−/−^ mice were exposed to DCs (1/500 in DMSO; 100,000 DCs) for 16 h and, after extensive washes, were cocultured with *i*NKT cell-enriched liver MNCs (500,000 cells) for 24 h. In this setting, IFNγ secretion is measured as a read-out for *i*NKT cell activity, although we cannot exclude that it can also be produced by other innate lymphocytes (e.g., NK cells) through bystander effect. Data represent the mean ± SEM of three independent experiments performed in duplicates. (C) Glycosphingolipids were extracted and isolated from CpG ODN-stimulated *B4galnt1*^−/−^ and *St3gal5*^−/−^ DCs, as described in the methods. Charged species were then separated from neutral species using DEAE ion-exchange chromatography prior to NP-HPLC. (D) Sialidase A-treated or untreated base-treated charged lipids extracted from CpG ODN-stimulated DCs were tested for *i*NKT cell antigenicity as in Fig 1B. Data represent the mean ± SEM of two independent experiments performed in triplicates. **P < 0.01; *P < 0.05. Underlying data used in the generation of this figure can be found in S1 Data. CpG, cytosine-phosphate-guanine; DC, dendritic cell; DEAE, diethylaminoethyl; MNC, mononuclear cell; IFNγ, interferon gamma; *i*NKT, invariant natural killer T; NK, natural killer; NP-HPLC, normal-phase high-pressure liquid chromatography; ODN, oligodinucleotide; WT, wild-type.

We previously reported that the activating *i*NKT cell Ag(s) that are present in DCs following TLR9 triggering was/were charged β-GlcCer derivative(s) [21]. Amongst the four biosynthetic pathways in vertebrates that stem from β-GlcCer, the ganglioside family contains most of the charged GSLs [22] (Fig 1A). In order to determine whether this class of GSLs was responsible for *i*NKT cell activation in our system, we used DCs generated from mice deficient in GA2/GM2/GD2/GT2 synthase (*B4galnt1*) or GM3 synthase (*St3gal5*), two key enzymes involved in ganglioside anabolism (Fig 1A). Cytosine-phosphate-guanine oligodinucleotide (CpG ODN)-stimulated DCs were used as a source of lipids for in vitro testing. Remarkably, while the lipid fraction from *B4galnt1*^−/−^ DCs, which lack all complex gangliosides, retained its capacity to activate *i*NKT cells, the lipid extract from *St3gal5*^−/−^ DCs failed to do so (Fig 1B). Normal-phase high-pressure liquid chromatography (NP-HPLC) analysis revealed that the activating fraction (CpG ODN-stimulated *B4galnt1*^−/−^ DCs) contains only two detectable species corresponding to the gangliosides GM3 (95%) and GD3 (5%), which are absent from the fraction isolated from CpG ODN-stimulated *St3gal5*^−/−^ DCs (Fig 1C). This suggested that the charged activating lipid(s) in CpG ODN-stimulated DCs is/are simple ganglioside(s). Since gangliosides are charged through incorporation of *N*-acetyl-neuraminic acid, we next tested the impact of sialidase treatment on the activity of the lipid(s). Interestingly, this treatment led to a significant reduction of its biological activity (Fig 1D). Altogether, these results indicate that the activating GSL(s) in TLR9-stimulated DCs include(s) simple sialylated ganglioside(s).

### TLR9 stimulation in DCs modulates ceramide moiety of simple ganglioside species

**Fig 2:**
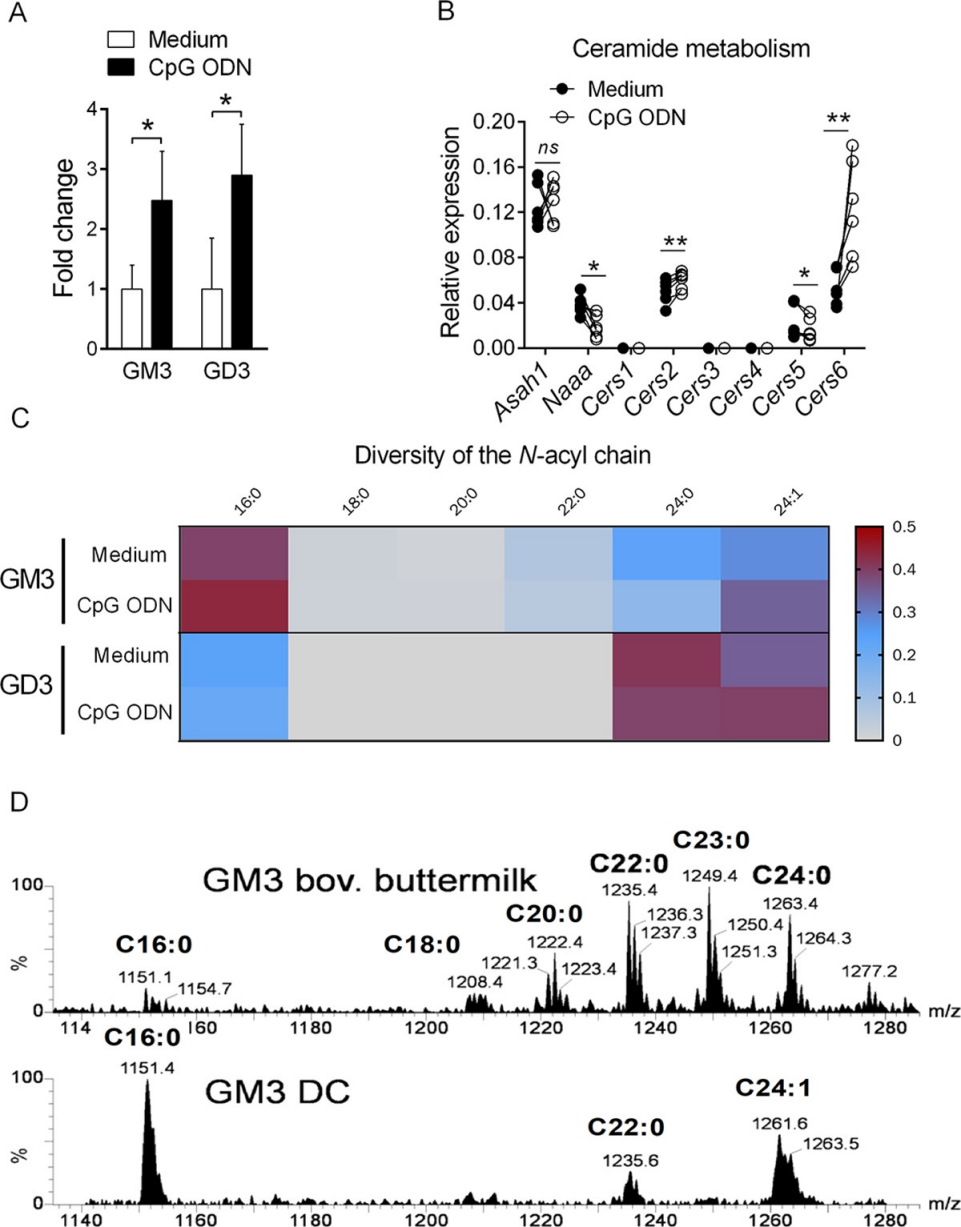
Modulation of ceramide and ganglioside metabolisms in DCs following CpG ODN treatment. (A) Increase of ES(-)-LC-MS/MS detected gangliosides GM3 and GD3 in CpG ODN-stimulated DCs shown as fold change relative to unstimulated cells (medium). Two sets of DCs were measured, and data are means ± SD. (B) RNAs from unstimulated or CpG ODN-stimulated DCs were harvested after 16 h stimulation, and *Asah1*, *Naaa*, and *Cers1-6* mRNA expression was measured by quantitative real-time PCR. Data represent paired individual values from six independent experiments. (C) Heatmap for DC *N*-acyl chain composition of GM3 and GD3 in unstimulated versus CpG ODN-stimulated cells measured by ES(-)-LC-MS/MS. Color code represents fractions of the total amount (1.0) of detected gangliosides, respectively. Two sets of DCs were measured and plotted as mean values. **P < 0.01; *P < 0.05. (D) ES(-)-LC-MS/MS analysis of the acyl chain composition of GM3 commercial bovine buttermilk and from mouse unstimulated DCs. Profiles of one experiment out of two are shown. Underlying data used in the generation of this figure can be found in S1 Data. Cers, ceramide synthase; CpG, cytosine-phosphate-guanine; DC, dendritic cell; ES(-)-LC-MS/MS, electrospray(-)-liquid chromatography-mass spectrometry/mass spectrometry, ns, not significant; ODN, oligodinucleotide.

In line with earlier studies [23], we previously suggested that TLR9 triggering in DCs resulted in metabolic changes in the general GSL pathway [21]. For instance, TLR9 engagement in DCs induced a modulation of gene expression in the GSL biosynthetic pathway, especially in key enzymes involved in the anabolism of simple gangliosides [21]. Accordingly, NP-HPLC analysis indicated that CpG ODN treatment led to a subtle increase of GM3 and GD3 in DCs (Fig 2A). Since the precise structure of the ceramide moiety is important for the antigenic properties of GSLs [8,14,24], we evaluated the composition of the lipid backbone of GM3 and GD3 contained in resting versus TLR9-stimulated wild-type (WT) DCs. As ceramide anabolism and catabolism is dependent, among others, on ceramide synthases (CerS) and ceramidases, respectively [19,25], we assessed the expression of these enzymes in DCs at the transcriptional level. The transcripts of two ceramidases (*Asah1* and *Naaa*) were detected in resting DCs. Of note, CpG ODN stimulation did not affect *Asah1* transcript expression, whereas it led to a significant reduction for *Naaa* expression (Fig 2B). In addition, we detected the mRNA expression of three ceramide synthases (*Cers2/5/6*) in resting DCs (Fig 2B) specialized in the synthesis of C16 (*Cers5* and *Cers6*) and C22/C24 (*Cers2*) ceramides [25]. Importantly, TLR9 triggering in DCs led to *Cers2* and *Cers6* up-regulation but *Cers5* down-regulation (Fig 2B). In line with this gene expression profile, identification of the *N*-acyl chain heterogeneity of GM3 and GD3 by mass spectrometry in resting DCs revealed the presence of various species differing in length and degree of unsaturation (Fig 2C). We detected the presence of at least six (assuming d18:1 sphingosine: C16:0, C18:0, C20:0, C22:0, C24:0, and C24:1) and three (C18:0, C24:0, and C24:1) species for GM3 and GD3, respectively, with variable abundance (Fig 2C). In line with our transcriptional analysis, TLR9 triggering led to a modulation in the proportion of specific ceramide forms. Specifically, CpG ODN stimulation preferentially favored C24:1 and C16:0 acyl chains in GM3, while reducing C22:0 and C24:0 species (Fig 2C). Meanwhile, CpG ODN led to an increased proportion of C24:1 acyl chain in GD3, paralleled with a reduction in C16:0 and C24:0 species (Fig 2C). Importantly, commercial gangliosides from bovine buttermilk, which have been widely used to test GSL antigenicity to *i*NKT cells, differ greatly in their *N*-acyl chain length composition compared to DC-derived GSLs (Fig 2D and S1 Table). For instance, C16:0 and C24:1 that are the main ganglioside species in mouse DCs are barely found in bovine buttermilk. On the other hand, C22:0, C23:0, and C24:0 gangliosides were abundant in bovine buttermilk, while they were minimally represented or undetectable in mouse DCs. Taken together, these results show that TLR9 stimulation induces not only increased production of GM3 and GD3 in DCs but also leads to an exquisite tuning of their ceramide backbone composition, both of which may have important biological consequences.

### Chemical synthesis of the C16:0 and C24:1 ganglioside species

**Fig 3:**
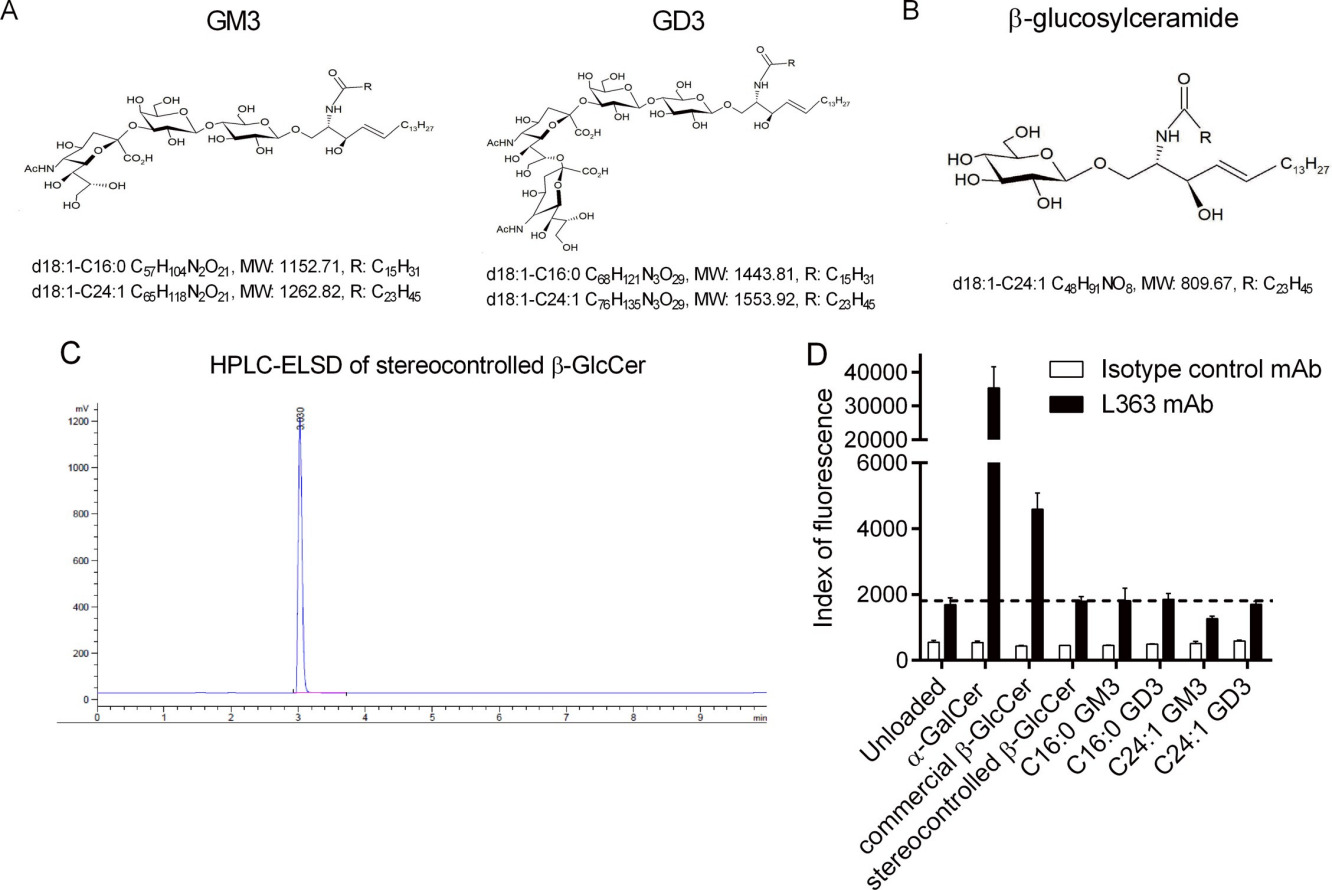
Structure and quality control of ganglioside species. (A) Structures and formulas of synthetic GM3 and GD3 gangliosides. (B) Structure and formula of synthetic β-glucosylceramide (C24:1). (C) HPLC-ELSD of stereo-controlled β-GlcCer. (D) Plate-bound CD1d dimers loaded with various glycolipids were incubated with the PE-conjugated L363 mAb. Fluorescence at 575 nm was measured using a microplate reader. One representative experiment out of two performed in triplicates is shown. Underlying data used in the generation of this figure can be found in S1 Data. β-GlcCer, β-glucosylceramide; CD1d, cluster of differentiation 1d; HPLC-ELSD, high-performance liquid chromatography-evaporative light scattering detector; mAb, monoclonal antibody; PE, phycoerythrin.

Based on these observations, we synthesized GM3 and GD3 with either a d18:1-C16:0 or a d18:1-C24:1 ceramide backbone (Fig 3A). The structures and synthetic schemes of the four gangliosides are illustrated in Supporting information (S1 Fig and S11–S14 Figs). With respect to the stereo-controlled construction of GM3 and GD3, we first produced a stereo-controlled β-GlcCer (Fig 3B and S15 Fig), which was then used as a building block for gangliosides assembly. β-GlcCer from commercial sources usually comes with α-contaminants (13, 14). On the contrary, NP-HPLC and hydrophilic interaction liquid chromatography-tandem mass spectrometry (HILIC-MS^2^) analyses indicate that there are no such contaminants in our stereo-controlled β-GlcCer compound (Fig 3C and S2 Fig), as compared to a synthetic mixture containing α anomers (S2 Fig). To demonstrate further the absence of α-hexosylceramide contaminants in our products, we probed, in a cell-free system, the structure of CD1d/synthetic GSL complexes using the L363 monoclonal antibody (mAb) that specifically recognizes CD1d/α-glycosylceramide interactions [14]. While it allowed detection of CD1d/α-GalCer and CD1d/commercial β-GlcCer complexes, L363 failed to bind to CD1d/ganglioside and CD1d/stereo-controlled β-GlcCer complexes (Fig 3D). Thus, we demonstrated the synthesis of β-linked ganglioside species for further *i*NKT antigenicity assessment.

### C24:1 gangliosides bind to CD1d

**Fig 4:**
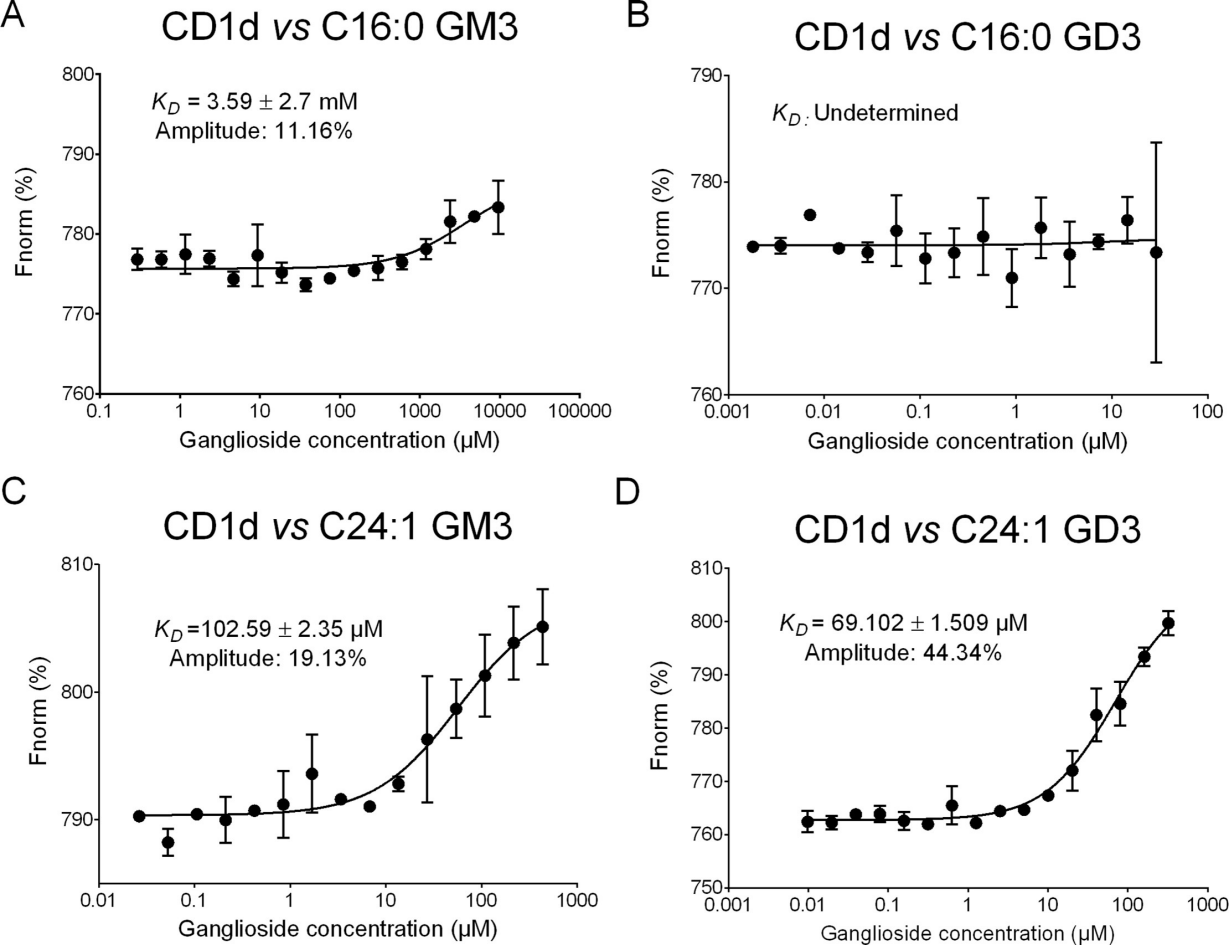
Thermophoretic analysis of NT647-labeled mouse CD1d-ganglioside interaction. Changes in thermophoresis of a titration of 125 nM NT647-labeled mouse CD1d with increasing concentrations of C16:0 GM3 (A), C16:0 GD3 (B), C24:1 GM3 (C), and C24:1 GD3 (D) are expressed as change of the normalized fluorescence (ΔFnorm = FHot/Fcold) and plotted. Line is a fit with Michaelis–Menten kinetics of ΔFnorm mean ± SD for each ligand concentration of three independent measurements. Underlying data used in the generation of this figure can be found in S1 Data.

Since previous crystallographic studies suggested that the length of the fatty acid chain influences the binding of lipids into the A′ and C′ pockets of CD1d molecules [26], we evaluated the capacity of synthetic GM3 and GD3 to bind to mouse CD1d molecules using microscale thermophoresis [27]. Surprisingly, d18:1-C16:0 GM3 and d18:1-C16:0 GD3 showed limited or no interaction with CD1d (Fig 4A and 4B). In contrast, C24:1 GM3 and C24:1 GD3 bound to CD1d with affinities of 102.5 ± 2.3 μM and 69.1 ± 1.5 μM, respectively (Fig 4C and 4D), which are approximately 70–100-fold weaker compared to CD1d/α-GalCer (C26:0) interactions. Of note, the neutral C24:1 β-GlcCer weakly interacted with CD1d (*K*_*D*_ = 2.54 mM) (S3 Fig) compared to C24:1 gangliosides, suggesting that additional hexose residues and/or charged residues significantly influenced the binding capacity of C24:1 GSLs into the CD1d groove. Taken together, our results demonstrate that C24:1 GM3 and C24:1 GD3 gangliosides are able to stably bind to mouse CD1d molecules.

### C24:1 gangliosides induce CD1d-dependent *i*NKT cell activation in vitro

**Fig 5:**
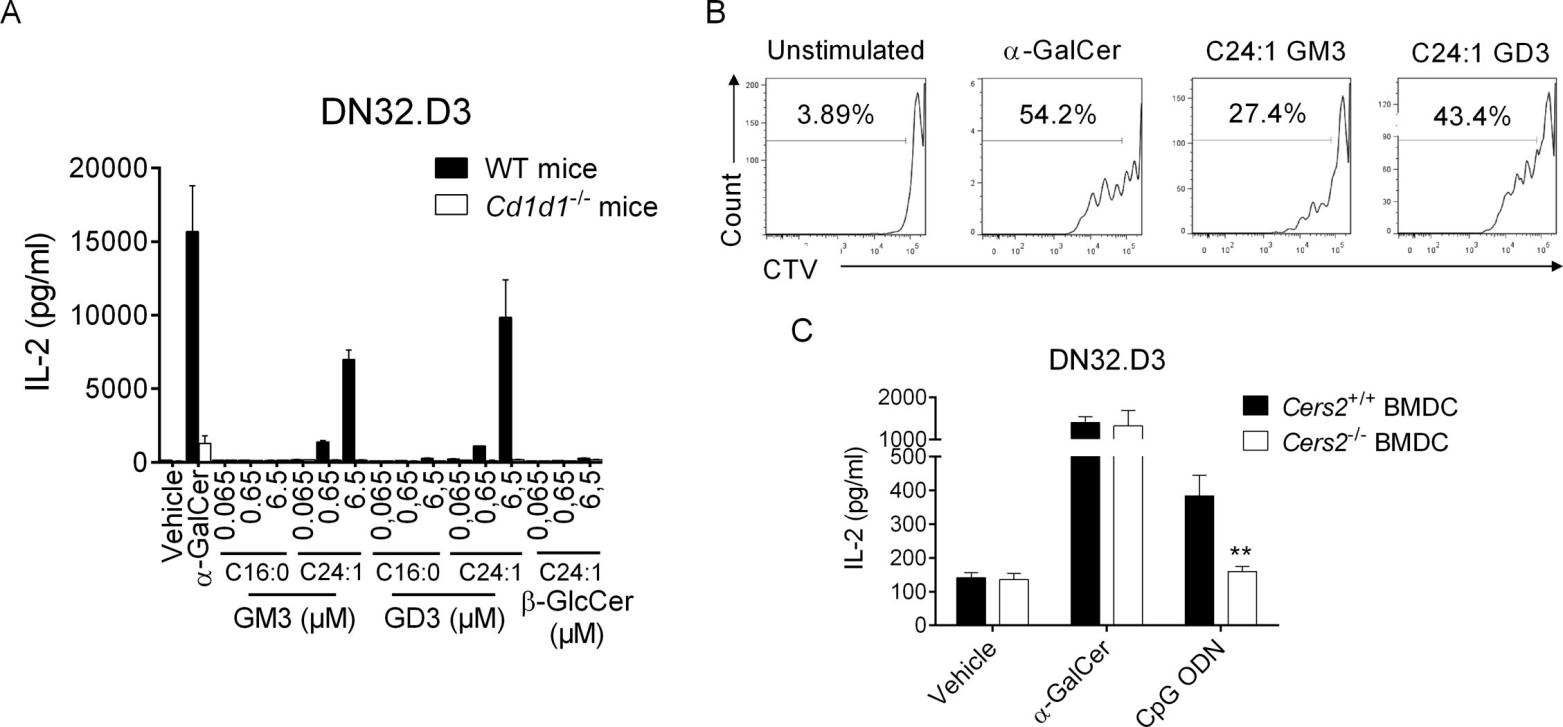
In vitro activity of gangliosides on *i*NKT cells. (A) DCs derived from WT or *CD1d1*^−/−^ mice were cultured with the mouse *i*NKT hybridoma DN32.D3 in presence of purified glycolipids at the indicated concentrations or vehicle in complete RPMI media for 24 h. IL-2 production was quantified by ELISA. Means ± SEM of one representative experiment out of three performed in triplicates are shown. (B) Cell Trace Violet-labeled spleen cells from C57BL/6 mice were cultured in absence or presence of α-GalCer (100 ng/ml), C24:1 GM3 (6.5 μM), or GD3 (6.5 μM) in complete RPMI plus recombinant IL-2. After 72 h, cells were harvested and analyzed by flow cytometry for CTV detection in live *i*NKT cells. Dot plots are representative of one experiment out of three. (C) DCs derived from *Cers2*^+/+^ or *Cers2*^−/−^ mice were cultured with DN32.D3 in presence of α-GalCer (20 ng/ml), CpG ODN (2 μg/ml), or vehicle in complete RPMI media for 24 h. Means ± SEM of IL-2 concentrations of one representative out of three experiments performed in duplicates are shown. **P < 0.01. Underlying data used in the generation of this figure can be found in S1 Data. α-GalCer, α-galactosylceramide; Cers, ceramide synthase; CpG, cytosine-phosphate-guanine; CTV, cell trace violet; DC, dendritic cell; IL-2, interleukin 2; *i*NKT, invariant natural killer T; ODN, oligodinucleotide; RPMI, Roswell Park Memorial Institute medium; WT, wild-type.

To assess ganglioside antigenicity, we cocultured DCs and *i*NKT cell hybridomas in the presence of synthetic GM3 and GD3. Both C24:1 GM3 and GD3 activated *i*NKT hybridomas in a CD1d- and dose-dependent manner, as measured by interleukin 2 (IL-2) production (Fig 5A and S4 Fig). In line with our affinity measurements, gangliosides bearing a C16:0 ceramide backbone showed little to no biological activity (Fig 5A). Consistent with a previous report [14], C24:1 β-GlcCer failed to activate *i*NKT cell hybridomas. Of note, *i*NKT cell hybridomas failed to respond to commercial gangliosides extracted from bovine buttermilk (S5 Fig). In addition, C24:1 gangliosides induced the proliferation of splenic *i*NKT cells (Fig 5B) but not conventional T cells in vitro (S6 Fig).

Since C24:1 ceramide biosynthesis is largely dependent on the activity of CerS2[28], we tested the ability of CpG ODN to uncover *i*NKT cell endogenous ligands in *Cers2*-deficient DCs. Interestingly, while CpG ODN-stimulated Cers2-competent DCs activated the *i*NKT cell hybridoma DN32.D3, *Cers2*-deficient DCs failed to do so (Fig 5C). As previously reported [29], it is noteworthy to mention that DCs from CerS2-null mice expressed reduced amounts of surface CD1d (approximately 50%) (S7A Fig). However, this only minimally affected their ability to present exogenous α-GalCer and C24:1 gangliosides to *i*NKT cell hybridomas (Fig 5C and S7B Fig). Collectively, these results indicate that C24:1 GM3 and C24:1 GD3 gangliosides are activating molecules for mouse *i*NKT cells in a CD1d-dependent manner.

### C24:1 ganglioside antigenicity requires intracellular machinery and is partially blocked by the L363 mAb

**Fig 6:**
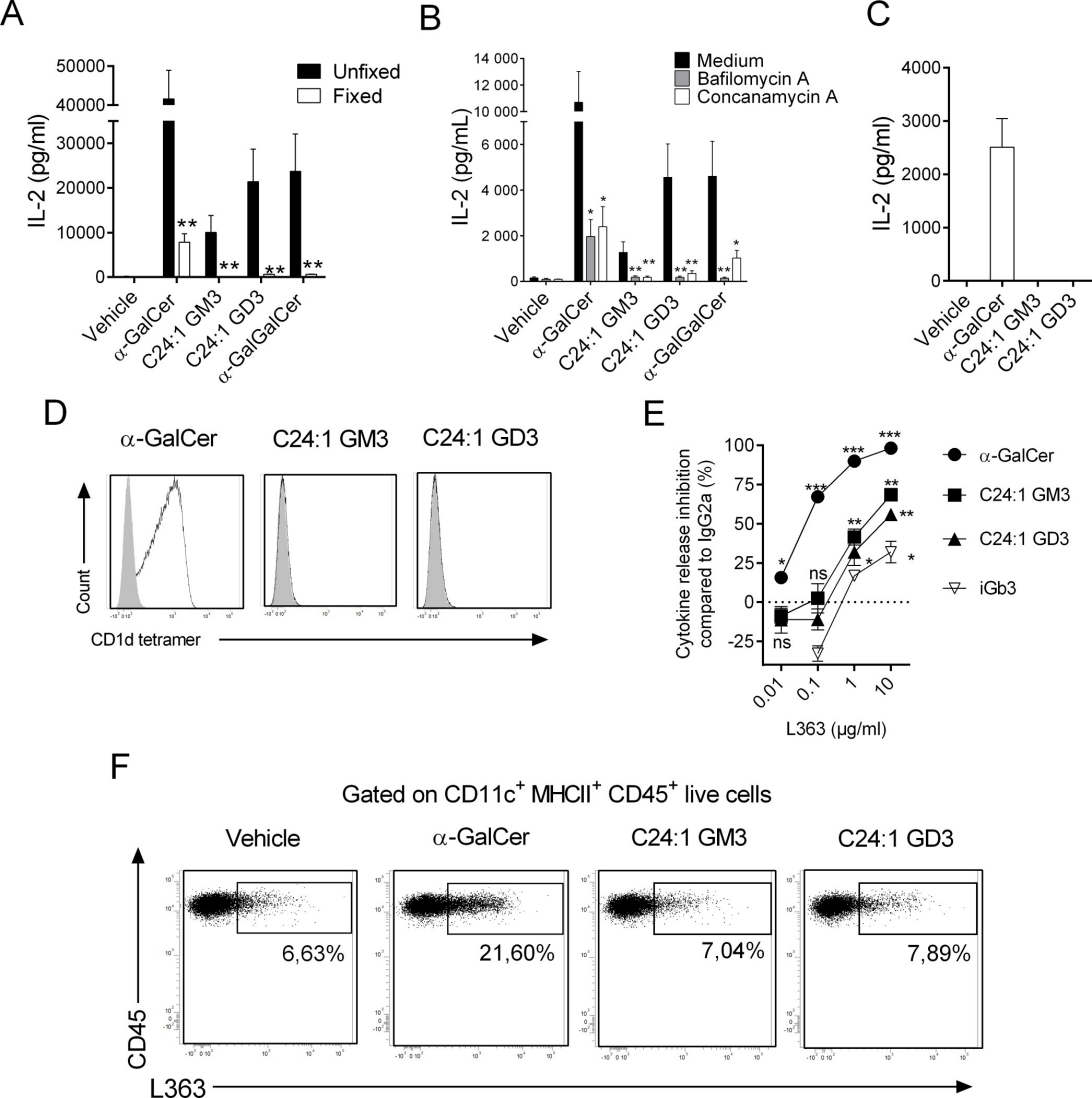
Influence of the DC intracellular pathway on the recognition of gangliosides by the *i*NKT TCR. (A) Fixed or unfixed DCs were cultured with the mouse *i*NKT hybridoma DN32.D3 in presence of purified glycolipids (23 pM for α-GalCer and 6.5 μM for gangliosides) or vehicle for 24 h. (B) Lysosomal acidification inhibitor–pretreated or not DCs were cultured with DN32.D3 in presence of glycolipids or vehicle. (C) Plate-bound CD1d dimers loaded with various glycolipids were cultured with DN32.D3 hybridoma for 24 h. (A–C) *i*NKT cell activity was evaluated as in Fig 5A. Data represent the means ± SEM of two experiments performed in triplicates. (D) DN32.D3 hybridoma was stained with PE-conjugated CD1d tetramers loaded or not with various glycolipids. Dot plots are representative of one experiment out of two. (E) DCs were cultured with DN32.D3 in presence of purified glycolipids (23 pM for α-GalCer, 1.5 μM for iGb3, and 6.5 μM for gangliosides) or vehicle for 24 h with increasing concentrations of L363 mAb or isotype control. NKT cell activity was evaluated as in Fig 5A. The dose-dependent L363 inhibition normalized to isotype control for each glycolipid is represented. Means ± SEM of two experiments performed in triplicates are shown. (F) Unloaded or glycolipid-loaded DCs were subjected to L363 mAb or Ig-control staining. Dot plots are representative of one experiment out of two for each condition. **P < 0.01; *P < 0.05. Underlying data used in the generation of this figure can be found in S1 Data. α-GalCer, α-galactosylceramide; CD1d, cluster of differentiation 1d; DC, dendritic cell; Ig, immunoglobulin; iGb3, isoglobotrihexosylceramide; *i*NKT, invariant natural killer T; mAb, monoclonal antibody; NKT, type I natural killer T cell; ns, not significant; PE, phycoerythrin; TCR, T-cell receptor.

As some GSLs require endosomal lipid transfer factors to be efficiently loaded onto CD1d [30,31], the importance of the intracellular pathway in antigenic capacity of C24:1 gangliosides was investigated. Glutaraldehyde fixation of DCs prior to culture severely impaired the capacity of gangliosides to activate DN32.D3, whereas α-GalCer partially retained its stimulating properties (Fig 6A). The response to α-Gal(α1–2)GalCer, a glycolipid that requires intracellular processing to reveal its antigenicity [24], was completely abolished by DC fixation (Fig 6A). In addition, the preincubation time of DCs with gangliosides strongly influenced their activating properties (S8 Fig). In the same vein, pretreatment of DCs with bafilomicyn A or concanamycin, two lysosomal acidification inhibitors, prior to ganglioside addition also abrogated *i*NKT cell activation in response to gangliosides (Fig 6B).

To test whether C24:1 gangliosides could be recognized in their native form by the *i*NKT TCR, DN32.D3 were cultured in presence of immobilized CD1d:ganglioside complexes. In this setting, we failed to detect any *i*NKT cell activation, as judged by cytokine release (Fig 6C). Moreover, mouse CD1d tetramers loaded with either GM3 or GD3 did not bind to *i*NKT cell hybridomas (Fig 6D), suggesting that the native compounds are not directly recognized by the *i*NKT TCR.

To assess whether intracellular processes such as hydrolysis and/or putative anomerization could convert β-linked gangliosides to α-linked GSLs, resulting in *i*NKT cell activation, we used L363 mAb in our assay. L363 mAb dose-dependently abolished DN32.D3 reactivity toward α-GalCer but only partly inhibited activity of gangliosides (approximately 50%–60% with the highest dose) (Fig 6E). Albeit at lower levels, it is noteworthy that iGb3, a self-GSL that activates *i*NKT cells when presented by CD1d in a β-conformation [32], was also significantly inhibited (approximately 20%) in the presence of high concentrations of L363 mAb (Fig 6E). Moreover, L363 binds to approximately 7% of resting DCs, suggesting the presence of small amounts of α-hexosylceramide:CD1d complexes at cell surface (Fig 6F). While α-GalCer loading increased L363 binding to DCs, gangliosides did not (Fig 6F). In conclusion, we suggest that gangliosides do not activate *i*NKT cells in their native forms and require the intracellular machinery to be presented into their antigenic conformation to the *i*NKT TCR.

### C24:1 gangliosides activate lung *i*NKT cells and partially protect mice against lethal pneumococcal infection

**Fig 7:**
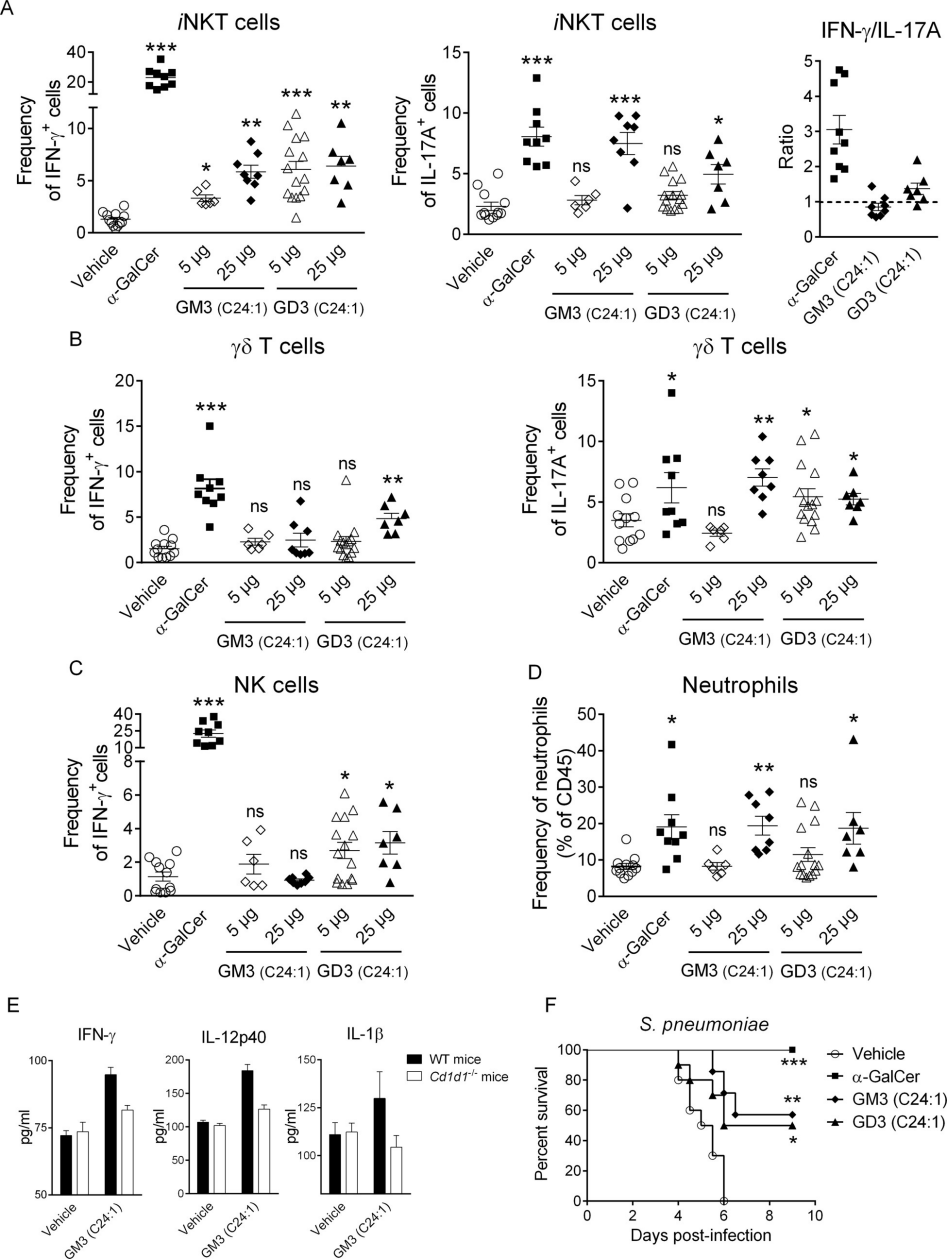
*i*NKT cell-dependent protective activity of C24:1 gangliosides on respiratory bacterial infection. (A) Lung *i*NKT cells from α-GalCer- or ganglioside-treated mice (12 h) were screened for intracellular IFNγ and IL-17 production. The ratio IFNγ-:IL-17A-producing *i*NKT cells is shown in the right panel. Lung γδT (B) and NK (C) cells were evaluated for intracellular IFNγ and IL-17 production as in Fig 7A. (D) Frequency of lung neutrophils (Live CD45^+^ Siglec F^-^ CD11b^+^ Ly6G^+^) in treated or untreated animals is shown. Individual values and means ± SEM of two independent experiments are shown (6–15 mice/group) for panel (A–D). (E) Levels of cytokines in response to C24:1 GM3 were measured in lung homogenates from WT or *Cd1d1*^−/−^ mice. Means ± SEM of two experiments are shown (4 mice/group). (F) Mice were pretreated with α-GalCer or gangliosides by the IN route one day prior to *S*. *pneumoniae* infection and monitored daily for survival (7–10 mice/group). ***P < 0.001; **P < 0.01; *P < 0.05. Underlying data used in the generation of this figure can be found in S1 Data. α-GalCer, α-galactosylceramide; γδT, gamma delta T; IFNγ, interferon gamma; IL-17, interleukin-17; IN, intranasal; *i*NKT, invariant natural killer T; NK, natural killer; WT, wild-type.

Activation of lung *i*NKT cells by intranasal inhalation of α-GalCer was shown to promote host defense mechanisms against respiratory bacterial (including pneumococcal) infections [33]. To evaluate ganglioside biological activity in vivo, we administered C24:1 GM3 and C24:1 GD3 intranasally. Interestingly, both lipids dose-dependently activated lung *i*NKT cells to produce interferon gamma (IFNγ) and IL-17A (Fig 7A). Unlike α-GalCer, ganglioside administration did not result in a strong T-helper (Th1)-biased response by *i*NKT cells, as assessed by IFNγ/IL-17A ratio (Fig 7A). Similar to α-GalCer, *i*NKT cell activation was accompanied by the transactivation of gamma delta (γδ)T cells and NK cells as well as the recruitment of neutrophils (Fig 7B–7D). In line with in vitro experiments, C16:0 ganglioside species’ inhalation failed to activate lung *i*NKT cells (S9 Fig). Importantly, CD1d blockade abrogated lung *i*NKT cell activation in response to C24:1 gangliosides (S10 Fig). Analysis of cytokine release in lung homogenates from WT and *Cd1d1*^−/−^ mice indicated that C24:1 ganglioside-induced immune response was dependent on the CD1d molecule (Fig 7E).

Finally, we investigated the effects of ganglioside inoculation on the host protective response against *Streptococcus pneumoniae*. As depicted in Fig 7F, all vehicle-treated mice died within 5–6 days following pneumococcal infection. In agreement with our previous findings [33], mice pretreated with α-GalCer did not present any observable clinical signs of disease and survived the infection. Interestingly, inoculation of synthetic GM3 or GD3 partially protected mice (around 50%) against lethal *S*. *pneumoniae*-induced pneumonia (Fig 7F). In sum, exogenous administration of C24:1 GM3 and C24:1 GD3 activates *i*NKT cells in vivo and protects against lethal respiratory bacterial infection.

## Discussion

Despite strong evidence suggesting the existence of self-lipid Ags for TCR-dependent *i*NKT cell selection and activation during inflammation, their precise nature remains unclear. Here, we have identified two particular species of the ganglioside family, which can be detected in TLR9-stimulated DCs, as molecules endowed with “indirect” antigenic capacities for *i*NKT cells. Synthetic d18:1-C24:1 β-linked GM3 and GD3 gangliosides can activate mouse *i*NKT cells in vitro and in vivo in a CD1d-dependent fashion. However, our data indicate that these molecules are not recognized in their native forms but require further intracellular changes to engage *i*NKT TCRs.

The contribution of the lipid moiety for *i*NKT cell antigenicity has mainly been explored with synthetic α-linked glycolipids. Nevertheless, some mammalian GSLs with particular ceramide structures present variable antigenic properties. For instance, mammalian α-GalCer, GlcCer, and sulfatide with a ceramide d18:1-C24:1 backbone have greater antigenic properties compared to other subspecies [8,14]. Similarly, despite sharing a similar polar head group, we observed that d18:1-C24:1 gangliosides activate *i*NKT cells, while their d18:1-C16:0 counterparts failed to do so, an effect which may be due to their low capacity to bind to mouse CD1d molecule. Our data also highlighted the importance of sialic acid residues for binding to CD1d. While both C24:1 GM3 and GD3 interact with CD1d with a *K*_*D*_ of μM range, the neutral C24:1 GlcCer presents a 10-fold lower affinity. Thus, the negative charge conferred by the sialic acids is likely to facilitate the loading into CD1d. At this stage, it remains unclear whether the sialic acid residue(s) participate(s) directly in the antigenicity of the compounds; however, we mainly observed in vitro and in vivo that GD3 had a higher activity on *i*NKT cells compared to GM3. While the use of *B4galnt1-* and *St3gal5-*deficient mice allowed us to suggest the activating capacity of GM3 and GD3, the existence of multiple charged ganglioside subspecies that may act as *i*NKT activators such as GM1, GM2, or GD1a cannot be definitely excluded.

It has been shown that some cancer cells contain gangliosides (GD3 and *N*-glycolyl-GM3) that can modulate *i*NKT cell activity [6,7,34]. In this context, the tumor Ag *N*-glycolyl-GM3 has been shown to interact with CD1d to induce moderate human *i*NKT cell proliferation[6]. GD3 antigenicity toward *i*NKT cells was only observed in preimmunized mice and was subset specific [7]. However, other studies have demonstrated that GD3 had inhibitory effects on TCR-dependent *i*NKT cell activation although confirming its binding to CD1d [35,36]. In these studies, authors have used bulk gangliosides (either purified or commercial), which may contain contaminants as well as various ceramide species, resulting in a potential competition between inhibitory and activating molecules. Moreover, we observed that C24:1 gangliosides are almost absent in commercial bovine buttermilk–derived GM3.

Amongst the high diversity of molecules bound to the CD1d molecules under steady state, it is interesting to note that GM3 could be eluted from both human and mouse CD1d, whereas GD3 was virtually undetectable [37,38]. However, the authors were only able to determine the structure for up to 25% of the total pool [37], which may explain the absence of GD3.

In line with previous studies [23,38], we observed that TLR triggering on DCs influences GSL abundance and diversity, including gangliosides. However, TLR-signaling ability to generate CD1d-restricted endogenous Ags is unlikely to be solely attributed to the neosynthesis of activating ligands. In fact, the time needed to generate new glycosphingolipids does not fit with the rapid response of *i*NKT cells. Here, we propose a concept in which TLR9 signaling affects ceramide metabolism to favor a pool of preexisting *i*NKT cell Ag precursors. Deciphering the respective contribution of C24:1 ceramide synthesis (de novo or salvage) versus catabolism of other species that may represent competitors for CD1d binding (C20:0-C24:0) in TLR9-dependent mechanism will be of the utmost importance. In addition, whether or not the triggering of other TLR members points to a similar mechanism will also need further investigation.

Our data also indicate a critical contribution for the intracellular machinery to efficiently present ganglioside to *i*NKT cell TCRs. Surprisingly, despite the lack of detectable α-GSL contaminants in our synthetic lipids, we observed that when used at high concentrations (μg/ml), the L363 mAb partially inhibited the antigenicity of both GM3 and GD3, suggesting a requirement for DC-derived α-glycosylceramides in these settings. Therefore, we can envision several nonmutually exclusive scenarios, including i) the involvement of putative specific endogenous anomerases, ii) the synergistic activity of endogenous α-glycosylceramides [14] with the C24:1 β-gangliosides, iii) the catabolism of gangliosides to simpler structure(s) with direct antigenic properties [39], or iv) that the *i*NKT TCRs reshape the primary β-linked sugar of the gangliosides into a new conformation that mimics α-linked glycolipids, as previously described for iGb3 [32]. The presence of traces of α-contaminants (below the threshold of our analytic procedures) in our synthetic lipids could also explain some of our findings, i.e., the inhibitory effect of L363 on ganglioside activity in our biological assay. However, while L363 is strongly active on α-GalCer at concentrations as little as 10 ng/ml, its effect on gangliosides is only observable with 100-fold higher concentrations. In addition, iGb3 activity is also partially inhibited with highest doses of L363 mAb. Interestingly, this observation can only be made in the coculture setting since L363 was neither able to bind immobilized CD1d:ganglioside complexes or ganglioside-pulsed DCs. Moreover, loading of CD1d tetramers with gangliosides did not allow detection of *i*NKT cells. Based on this, we believe that this finding is unlikely due to contamination but rather to a certain degree of nonspecificity for the L363 mAb against β-GSLs or to a yet-to-be-defined biological phenomenon, as stated above.

Can GM3 and/or GD3 represent self-Ags during *i*NKT cell development? Porubsky and colleagues demonstrated that *St3gal5*^−/−^ and *St8sia1*^−/−^ mice present a normal *i*NKT cell compartment [40]. Thus, simple gangliosides do not appear to be critical in *i*NKT cell positive selection or maturation/differentiation. However, in line with its involvement in *i*NKT cell antigenicity, we recently demonstrated the critical involvement of CerS2, an enzyme involved in the generation of very long–chain sphingolipids, including d18:1-C24:1 GSLs in *i*NKT cell development and survival [29]. Globally, ceramide synthases and ceramidases have been shown to display variable substrate specificity. In this context, a tight regulation of ceramide metabolism could be critical in either favoring or hindering self-*i*NKT cell Ag formation. Thus, a better characterization of the role of enzymes of the ceramide metabolism including other families such as β-Glucocerebrosidase, sphingomyelinases, and hexosaminidases in *i*NKT cell development and/or activation is warranted.

Since the level of expression of endogenous Ags must be low and tightly controlled under homeostatic conditions to prevent *i*NKT cell autoreactivity and development of autoinflammatory processes, the uncovering of gangliosides as *i*NKT cell activators makes sense. Indeed, while native gangliosides do not present direct antigenic properties on *i*NKT cells, this preexisting pool could be rapidly converted into bioactive Ags, resulting in rapid response of *i*NKT cells upon stressful conditions. Furthermore, gangliosides do not have a strictly host origin. Lipid-enriched diet might represent an exogenous source of gangliosides. Given the role of *i*NKT cells in diet-induced obesity and insulin resistance [41,42], the contribution of dietary gangliosides in these diseases should be considered. In line with this, GM3 has been proposed to participate in insulin resistance in genetic models of diabetes [43]. Gut microbiota has been proposed to negatively regulate *i*NKT cell number and functions [44], questioning the putative involvement of gangliosides in this mechanism. Since gangliosides have never been reported to be synthesized/produced by commensals, they are unlikely to directly contribute to this phenomenon. It is also worth noting that gangliosides are overexpressed in human milk with GM3/GD3 as major species in colostrum and GM3 in mature milk [45]. Since human *i*NKT cells developing during fetal life can mature in the small intestine [46], encounters with milk-derived gangliosides shortly after birth may provide positive signals required in this process. A recent study elegantly demonstrated the presence of active α-linked monohexosylceramides Ags in bovine milk that could also play a part in these mechanisms [17]. Hence, the putative presence of active α-linked gangliosides would also be worth investigating. Finally, since we and others [45] observed that bovine and human milk have strong differences in ganglioside species, a comparative analysis of the *i*NKT cell compartment in breast-fed versus bottle-fed infants would be worthy of study.

In conclusion, our study reinforces the current concept that “innate-like” T lymphocytes are able to sense fine modulation in cellular metabolism to become activated. We demonstrate how an innate signal can be detected by *i*NKT cells through subtle alterations of the ceramide and/or ganglioside metabolism and the generation of specific antigenic self-lipids.

## Methods

### Ethics statement

All experiments were conducted on C57BL/6J genetic background mice, and the performance was in compliance with current national and institutional regulations and ethical guidelines (B59-350009) and approved by the Comité d’Ethique en Experimentation Animale Nord-Pas de Calais (C2EA-75) under the protocol number 2015121722376405. All efforts were taken to minimize mouse usage to maximize necessary results; provide the best veterinary care; and minimize discomfort, distress, and surgery with anesthetic procedures and euthanasia. Euthanasia was performed using a lethal injection of pentobarbital. In survival experiments, mice were euthanized when reaching one of these endpoints: dehydration, loss of ability to ambulate, labored respiration, or weight loss (>20%).

### Mice

8- to 12-week-old male WT C57BL/6J mice were purchased from Janvier (Le Genest-St-Isle, France). The generation of *Cd1d1*^−/−^ C57BL/6J mice has been previously described [47]. Mice deficient of *B4galnt1* (EC 2.4.1.92) and *St3gal5* (EC 2.4.99.9) were provided by R. Proia (National Institutes of Health, Bethesda, MD, United States of America). The generation of CerS2 null mice has been described in [28]. Mice were bred in our own facility in specific pathogen-free conditions. For *S*. *pneumoniae* infection, mice were maintained in a biosafety level 2 facility.

### Reagents and abs

Type B CpG ODN (ODN 1826) was from Cayla (Toulouse, France). α-GalCer was produced in house. Glyko Sialidase A was from PROzyme (Hayward, CA, USA). mCD1d protein was purchased from Interchim (Montluçon, France). Recombinant Soluble Mouse CD1d:Ig Fusion Protein (CD1d dimer XI) was from BD Biosciences (Le Pont de Claix, France). Cell Trace Violet Cell Proliferation Kit was from ThermoFischer scientific. Anti-CD1d (19G11) and its isotype control (LTF-2, Rat IgG2b) were from Bio X Cell (West Lebanon, NH, USA). Bovine buttermilk GM3 and GD3 were from Biovalley (Nanterre, France). The commercial d18:1-C24:1 β-GlcCer was from Interchim. The commercial α/β-GlcCer-mix (15/85) was from Avanti Polar Lipids. α-GalGalCer and iGb3 were kindly provided by Dr. Steven Porcelli (Albert Einstein College of Medicine, New York City, NY, USA). L363 (IgG2a) mAb (purified or PE-conjugated) and isotype control were from eBiosciences (San Diego, CA, USA). PBS-57 glycolipid-loaded and unloaded control CD1d tetramers (APC- or PE-conjugated) were from the National Institute of Allergy and Infectious Diseases Tetramer Facility (Emory University, Atlanta, GA, USA). Monoclonal antibodies against mouse CD45 (APC-Cy7-conjugated), CD3 (Pacific Blue- or PerCP-Cy5.5–conjugated), TCRδ (PerCP-Cy5.5-conjugated), NK1.1 (PE-Cy7- or FITC-conjugated), Ly6G (FITC-conjugated), CD11b (PerCP-Cy5.5–conjugated), IFNγ (AF647-conjugated), IL-17A (PE-conjugated), and appropriated isotype controls were purchased from BioLegend (San Diego, CA, USA) and BD Pharmingen. Mouse ELISA kits are from R&D systems (Minneapolis, MN, USA) and eBioscience.

### Generation of BMDCs, lipid extraction/purification, sialidase treatment, and glycolipid analysis

Briefly, bone marrow (BM) precursors from various gene-targeted mice were cultured in complete IMDM medium supplemented with 10% FCS and 1% of supernatant from a granulocyte-macrophage colony-stimulating factor (GM-CSF)–expressing cell line (J558-GM-CSF) for 14 days. BMDCs (>90% purity) were stimulated or not with CpG ODN (2 μg/ml) for 16 h. BMDCs were then collected and dry pellets were frozen (−20°C) until further treatment/analysis.

Lipids were extracted from resting or CpG ODN-stimulated DCs, as previously described [21]. To remove all terminal sialic acid residues, lipids were dried under nitrogen and resuspended in 50 mM sodium acetate, 1 mg/ml sodium taurodeoxycholate, pH 5.5. N-acetylneuraminate glycohydrolase (sialidase, EC 3.2.1.18, *Arthrobacter ureafaciens* recombinant expressed in *E*. *coli*), 50 mU, was added for 48 h in a total volume of 20 μl. All lipid fractions were desalted over C18 cartridges (pre-equilibrated with 2 x 1 ml methanol and 2 x 1 ml water). The volume of digest was made up to 100 μl with water and then the sample was applied to the cartridge; the sample tube was washed in 2 x 100 μl chloroform: methanol: water 1:2.2:1 (v/v/v) and applied to the cartridge. The cartridge was then washed with 3 x 1 ml water and the lipids were eluted with 1 x 1 ml chloroform: methanol 98:2 (v/v), 2 x 1 ml chloroform: methanol 1:3 (v/v), and 1 x 1 ml methanol and mixed thoroughly. Purified lipid fractions were dried under nitrogen for in vitro testing after validating the digestion by subjecting 0.5% of the lipid fraction to ceramide glycanase digestion, anthranilic acid labeling, and NP-HPLC analysis, as previously described [48].

### RNA extraction, cDNA synthesis, and real-time PCR

Total RNA from resting or CpG ODN-treated DCs were isolated with the Nucleospin RNA Plus extraction kit (Macherey-Nagel, Hoerdt, France), and cDNA were synthesized from 1 mg of total RNA with random hexamer primers and Superscript III (Invitrogen, Cergy Pontoise, France) according to standard procedures. cDNAs were used as templates for PCR amplification with the SYBR Green PCR Master Mix (Molecular Probes, Leiden, the Netherlands) and the ABI PRISM 7700 Sequence Detector (Applied Biosystems, Foster City, CA). Primers, which are listed in S2 Table, were designed by the Primer Express Program (Applied Biosystems) and used for amplification in triplicate assays. PCR amplification of *Gapdh* was performed to control for sample loading and to allow normalization between samples. ΔCt values were obtained by deducting the raw cycle threshold (Ct values) obtained for *Gapdh* mRNA, the internal standard, from the Ct values obtained for investigated genes. For graphical representation, data are expressed as relative expression of mRNA levels.

### Analysis of the structure of GM3 and GD3 ceramide tail

Aliquots corresponding to 20,000 BMDCs/μL were mixed with internal lipid standards for analysis by LC MS/MS using an Aquity I-class UPLC and a Xevo TQ-S “triple-quadrupole” instrument, both from Waters. Using a CORTECS HILIC column (2.1 mm x 100 mm; 1.7 μm, Waters), gangliosides were measured in negative mode with a gradient between 80% solvent A (90% acetonitrile) and 100% solvent B (50% acetonitrile), both containing 10 mM ammonium formate as additive. Gangliosides were analyzed with the MS/MS-transitions [GM3 − H]− > [NeuAc − H]− and [GD3 − 2H]2− > [NeuAc − H]− by multireaction monitoring (MRM) at optimized collision energies of 50 eV and 30 eV, respectively. Transitions reflect by majority GM3/GD3 species with d18:1 long chain base (C18-sphingosine) and C16 to C24 fatty acyl chain length, as C18-sphingosine is the dominant sphingoid base. GM3 (d18:1; C19:0) was used as internal standard. Qualitative measurements in positive mode were performed with the MS/MS-transitions [GM3 + H]+ > [Sph(d18:1 + H − 2H2O]+ and [GM3 + H]+ > [Sph(d20:1 + H − 2H2O]+ by multireaction monitoring (MRM).

### Chemical synthesis of glycosphingolipids

The general synthesis of GM3 and GD3 are based on the strategies reported by Akira Hasegawa [49] and Tomoya Ogawa [50]. Synthesis of GD3 (S11 Fig) began with regioselective glycosylation of lactosyl diol (2) with dimeric thioglycoside (1) using *N*-iodo-succinimide (NIS) and triflic acid (TfOH) as promoter at −25°C, affording sole tetrasaccharide (3) containing α-sialyl-(2→8)-sialic acid unit α-glycosidically linked to O-3 of D-galactose residue in the oligosaccharide chains. The tetrasaccharide (3), after palladium on carbon catalyzed hydrogenation and O-acetylation, was converted into tetrasaccharide (4). Further desilylation and anomeric hemiacetal activation gave trichloroacetimidate (5), which was coupled with either d18:1-C16:0 ceramide (6) or d18:1-C24:1 Ceramide (7), giving glycosyl ceramides (8a) or (8b), respectively. Theses glycosides were then transformed via global deacetylation and hydrolysis of methyl esters into GD3. The general synthesis of GM3 followed a similar strategy (S12 Fig). Briefly, thioglycoside (9) was regioselectively coupled with lactosyl diol (2) to generate trisacchride (10). Sequential hydrogenation and O-acetylation of (10) gave peracylated compound (11), which was further subject to desilylation to afford hemiacetal in proximal sugar. By treatment with trichloroacetonitrile, the obtained hemiacetal was converted into Schmidt donor (12), which was then coupled with either d18:1-C16:0 Cer (6a) or d18:1-C24:1 Cer (7a) to afford (13a) or (13b), respectively. These protected triglycosylceramides were each transformed into the targeted gangliosides GM3 via O-deacetylation and saponification of the methyl ester.

A stereo-controlled synthetic route was adopted for the production of GD3 and GM3 (S13 and S14 Figs) in pure *β* form according to the report by Shunichi Hashimoto [51]. This strategy features coupling of trisaccharide (22) or disaccharide (27) with a stereocontrolled building block (17), which excludes possibility of *α* isomer contamination. In this regard, a disarmed donor (14) was chosen in the glycosylation reaction to form *β*-glycoside in terms of neighboring participation effect, and β-glycosylceramide was thus prepared (S15 Fig). Removal of chloroacetyl protecting group of 16 gave acceptor (17), which was used as generic acceptor for the assembly of GD3 and GM3 subsequently. Compound 17 was further subject to saponification and Birch reduction to afford pure *β* GlcCer. Assembly of stereo-controlled GD3 tetrasaccharide was implemented by coupling of (17) with (22), followed by global deprotection and reduction. The stereo-controlled synthesis of GM3 was completed in the same fashion as stereo-controlled GD3. The stereo-controlled GD3 and GM3 matched up with GD3 and GM3 synthesized with general method by ^1^HNMR spectra comparison (S16 and S17 Figs). Comparison of controlled GD3 or GM3 with GD3 or GM3 made with conventional method in biological assays using type I natural killer T cell (NKT) hybridomas indicated no differences in their antigenic capacities. Separation and identification of α- and β-GlcCers was conducted with the recently published HILIC-MS^2^ method [15]. ^1^H NMR, HPLCs, and LC-MS profiles of synthesized GD3 and GM3 are included in (S18–S21 Figs). Synthetic chemistry of gangliosides will be published separately.

### Microscale thermophoresis

Quantitative analysis of the interaction between mCD1d and glycolipids was performed by Microscale Thermophoresis (MST) using a Monolith NT115 instrument (NanoTemper Technology GmbH Munich, Germany). Recombinant mCD1d protein (MW = 33.7 kDa) was labeled with a reactive RED dye (NT-647) by N-hydroxysuccinimide (NHS) coupling (NanoTemper red-NHS kit) following the manufacturer's protocol. Briefly, mCD1d (15 μM) was mixed with the dye (45 μM) and incubated for 30 min in the labeling buffer (130 mM NaHCO_3_, 50 mM NaCl, pH 8.2) at room temperature. Unincorporated dye was removed by gel filtration with a Sephadex G-25 column, and mCD1d was finally collected in PBS 25 mM, 0.05% Tyloxapol to a final concentration of 2.5 μM. The thermodynamic affinity constant characterizing the molecular interaction of mCD1d with each analyzed glycolipid was determined by performing a titration of the corresponding nonfluorescent glycolipids against a constant concentration of NT-647-labeled mCD1d (125 nM). Briefly, C16:0 GM3, C24:1 GM3, C16:0 GD3, C24:1 GD3, and C24:1 β-GlcCer were solubilized in DMSO. A 10-fold factor dilution of these stock solutions was performed in PBS 25 mM, 0.05% Tyloxapol to reach a final maximal proportion of DMSO equal to 5%. Thus, titration of each glycolipid was performed by serial dilutions of these solutions in PBS 25 mM, 0.05% Tyloxapol to which an equal volume of NT-647-labeled mCD1d was added. Then, reaction mixture was loaded into Premium capillaries and subsequently analyzed by MST using 60% MST power with a laser-on time of 30 sec per samples and an intensity of the light-emitting diode (LED) of 20%. Fluorescence time trace for each glycolipid concentration was recorded for each interaction. All analysis was performed in triplicate by using the NanoTemper MO.affinity analysis software version 2.1 and thermodynamic dissociation constant (*K*_*D*_) characterizing the molecular interaction between mCD1d, and each glycolipid was determined by plotting the temperature-dependent change of the normalized fluorescence (ΔFnorm = F_Hot_/F_cold_, with F_cold_ = fluorescence intensity before the IR-Laser is on [area marked in blue], and F_hot_ = fluorescence intensity 1 sec before the laser is off [area marked in red]) with the corresponding concentration of each unlabeled glycolipid. The resulting binding curves were fitted using a 1:1 binding model to determine the average *K*_*D*_ values.

### iNKT cell activation assays

To investigate *i*NKT cell reactivity, 1 x 10^5^ BMDCs were cultured with 1 x 10^5^ mouse *i*NKT hybridomas DN32.D3 and/or 2C12 in presence of CpG ODN, purified glycolipids or vehicle in complete RPMI media supplemented with 5% FCS for 24 h. In some cases, neutralizing or control Abs were added during the coculture. To fix DCs, cells were exposed to glutaraldehyde (0.05% in PBS) for 3 min and then extensively washed. To investigate the activity of lipids extracted from DCs, 1 x 10^5^ DCs were exposed to lipid fractions (1/500 of total extracts from 5 x 10^7^ BMDCs) or vehicle alone for 16 h, washed, and then cocultured with liver MNCs (5 x 10^5^) in the presence or absence of recombinant mIFNβ (1000 U/ml). Coculture supernatants were collected, and cytokine production was measured by ELISA (R&D Systems).

### Identification of α-monohexosylceramide: CD1d complexes

To probe the presence of α-monohexosylceramides in our synthetic compounds, glycolipids were loaded on CD1d dimers for 16 h (at a 200 molar excess of lipids except for α-GalCer that was loaded at a 20 molar excess) in presence of 0.5% of Tyloxapol. Then, CD1d:glycolipid complexes were coated on a flat bottom 96-well plate for 4 h in presence of PE-labelled L363 mAb or appropriate Ig control. Fluorescence at 578 nM was measured on a microplate reader TECAN infinite (Männedorf, Switzerland). To test the presence of α-monohexosylceramide:CD1d complexes in ganglioside-loaded DCs, WT-derived BMDCs were loaded for 16 h with the various synthetic gangliosides (6.5 μM), extensively washed, and labeled with the L363 mAb or Ig control. α-GalCer (0.2 nM) was used as a positive control.

### Loading of CD1d tetramers with synthetic glycolipids

Unloaded CD1d tetramers were loaded for 16 h with a 5 (α-GalCer) or 50 (gangliosides) molar excess of glycolipids in presence of 0.5% of Tyloxapol. Unloaded or loaded CD1d tetramers were then probed against NKT hybridomas.

### Preparation of lung leukocytes and flow cytometry

Mice were IN injected with α-GalCer (500 ng/mouse) or ganglioside species (5 or 25 μg/mouse) in 50 μl of saline. In some cases, mice were IP pretreated (4 h) with an anti-CD1d mAb (19G11; 500 μg/mouse) or its isotype control (LTF-2). Twelve hours later, lungs were harvested, and leukocyte suspensions were prepared by classical procedures. Briefly, perfused lungs were harvested and finely minced in a Petri dish. Then, lung pieces were enzymatically digested (20 min at 37°C) in saline containing 1 mg/ml of collagenase type VIII (Sigma-Aldrich) and 1 μg/ml of DNase type I (Roche). After washes, pellets were resuspended in a 20% Percoll gradient and centrifuged (2,000 rpm at RT for 15 min). Cells in pellet were collected and washed in PBS containing 2% FCS. Erythrocytes were removed using a red blood cell lysis buffer (Sigma-Aldrich). Lung mononuclear cells were incubated in complete RPMI 1,640 medium in presence of Golgi Plug/Golgi Stop (BD Biosciences) (2 h at 37°C). After washes, cells were labelled with the appropriate dilutions of the various mAbs (30 min at 4°C) in PBS containing 2% FCS. Cells were then washed, and fixed using an intracellular fixation buffer (eBioscience, CliniSciences, Montrouge, France). Next, fixed lung mononuclear cells were treated with a permeabilization buffer (eBioscience) according to the manufacturer’s instructions. Cells were stained with anti-IFNγ and anti-IL-17A mAbs or corresponding isotype controls and acquired on a Fortessa (Becton Dickinson, Rungis, France) cytometer. Analyses were performed using the FlowJo software (Treestar, OR, USA).

### Inoculation of glycolipids and infection with S. pneumoniae

*S*. *pneumoniae* serotype 1 clinical isolate E1586 sequence type ST304 has been described elsewhere [33]. Mice were anesthetized and administered IN with 50 μl PBS containing live bacteria (1 x 10^6^ cfu). Twelve hours prior infection, mice were IN injected with 500 ng of α-GalCer (0.58 nmol) or 25 μg of gangliosides (20 nmol for GM3 and 16 nmol for GD3) in 40 μl of saline. Then, mice were monitored daily for illness and mortality for a period of 9 days.

### Statistical analysis

Results are expressed as the mean ± SEM. All statistical analysis was performed using GraphPad Prism software (San Diego, CA, USA). The statistical significance was evaluated using nonparametric (paired or unpaired) Mann–Whitney U or Kruskal–Wallis (followed by a Dunn’s post-test) tests to compare the means of biological replicates in each experimental group. Survival rates after *S*. *pneumoniae* challenge were analyzed using a log-rank test. Results with a *P* value of less than 0.05 were considered significant. ns, not significant; *P < 0.05; **P < 0.01; ***P < 0.001.

## Supporting information

S1 Fig Structures of gangliosides. (TIF)

S2 Fig HILIC-MS^2^ analyses of stereo-controlled β-GlcCer. HILIC-MS^2^ of stereo-controlled β-GlcCer and an α/β-GlcCer mixture. α/β-GlcCer, α/β-glucosylceramide; HILIC-MS^2^, hydrophilic interaction liquid chromatography-tandem mass spectrometry.
(TIF)

S3 Fig Thermophoretic analysis of NT647-labelled mCD1d-β-GlcCer interaction. Changes in thermophoresis of a titration of 125 nM NT647-labeled mCD1d with increasing concentrations of C24:1 β-GlcCer are expressed as change of the normalized fluorescence (ΔFnorm = FHot/Fcold) and plotted. Line is a fit with Michaelis–Menten kinetics of ΔFnorm mean ± SD for each ligand concentration of three independent measurements (*n* = 3). β-GlcCer, β-glucosylceramide.
(TIF)

S4 Fig Reactivity of the 2C12 *i*NKT cell hybridoma towards synthetic gangliosides. DCs derived from WT mice were cultured with the mouse *i*NKT hybridoma 2C12 in presence of purified glycolipids at the indicated concentrations or vehicle in complete RPMI media supplemented with 5% FCS for 24 h. *i*NKT cell response was judged based on IL-2 production. Means ± SEM of one representative experiment out of two performed in triplicates are shown (*n* = 2). DC, dendritic cell; FCS, fetal calf serum; IL-2, interleukin 2; *i*NKT, invariant natural killer T; RPMI, Roswell Park Memorial Institute medium; WT, wild-type.
(TIF)

S5 Fig Bovine buttermilk-derived gangliosides do not activate *i*NKT cells. DCs derived from WT mice were cultured with the mouse *i*NKT hybridoma DN32.D3 in presence of α-GalCer (20 ng/ml), bovine buttermilk GM3 (10 μM) and bovine buttermilk GD3 (10μM), or vehicle in complete RPMI media supplemented with 5% FCS for 24 h. *i*NKT cell response was measured based on IL-2 production. Means ± SEM of one representative experiment out of three performed in triplicates are shown. α-GalCer, α-galactosylceramide; DC, dendritic cell; FCS, fetal calf serum; IL-2, interleukin 2; *i*NKT, invariant natural killer T; RPMI, Roswell Park Memorial Institute medium; WT, wild-type.
(TIF)

S6 Fig C24:1 GM3 and C24:1 GD3 do not induce splenic conventional T cell proliferation. CTV-labeled spleen cells from C57BL/6 mice were cultured in absence or presence of α-GalCer (100 ng/ml), C24:1 GM3 (6.5μM), or GD3 (6.5μM) in complete RPMI plus IL-2. After 72 h, cells were harvested and analyzed by flow cytometry for CTV detection in live conventional αβT cells (CD45^+^ TCRβ^+^ CD1d tetramer^−^). Dot plots are representative of one experiment out of three. α-GalCer, α-galactosylceramide; CTV, cell trace violet; IL-2, interleukin 2; RPMI, Roswell Park Memorial Institute medium; TCR, T-cell receptor.
(TIF)

S7 Fig Expression and Ag presentation capacity of CD1d on DCs from Cers2 null mice. (A) Representative FACS plot of BMDCs from Cers2^+/+^ and Cers2^−/−^ mice (gated on live CD45^+^ cells) is shown in the left panel. Histogram for CD1d surface expression on DCs of *Cers2*^+/+^ and *Cers2*^−/−^ mice is shown in the right panel. Isotype controls for *CerS2*^+/+^ and *CerS*2^−/−^ are shown in red and blue, respectively. These data are representative of three independent experiments. (B) DCs derived from Cers2^+/+^ or Cers2^−/−^ mice were cultured with the mouse *i*NKT hybridoma DN32.D3 in presence of glycolipids at the indicated concentrations or vehicle for 24 h. *i*NKT cell response was judged based on IL-2 production. Means ± SEM of three experiments are shown. BMDC, bone marrow-derived dendritic cell; Cers, ceramide synthase; DC, dendritic cell; FACS, fluorescence-activated cell sorting; IL-2, interleukin 2; *i*NKT, invariant natural killer T.
(TIF)

S8 Fig Pulse timing on DCs influences ganglioside activity on *i*NKT cell hybridomas. DCs derived from WT mice were pulsed with C24:1 GM3, C24:1 GD3, or vehicle for the indicated amount of time. Then, cells were extensively washed and cultured with the *i*NKT cell hybridoma DN32.D3 for 24 h. *i*NKT cell response was judged based on IL-2 production. Means ± SEM of two experiments performed in triplicates are shown. DC, dendritic cell; IL-2, interleukin 2; *i*NKT, invariant natural killer T; WT, wild-type.
(TIF)

S9 Fig Instillation of C16:0 GD3 does not lead to lung *i*NKT cell activation. Lung *i*NKT cells from vehicle- or C16:0 GD3-treated mice (12 h) were screened for intracellular IFNγ and IL-17A production. Individual values and means ± SEM from two independent experiments are shown (5 mice/group) for panel. Underlying data used in the generation of this figure can be found in S1 Data. IFNγ, interferon gamma; IL-17A, interleukin-17A; *i*NKT, invariant natural killer T.
(TIF)

S10 Fig CD1d blockade abrogates lung *i*NKT cell activation upon C24:1 ganglioside instillation. Lung *i*NKT cells from vehicle-, α-GalCer- or C24:1 GM3-treated mice (12 h) were screened for intracellular IFNγ production. Mice were intraperitoneally pretreated (4 h prior glycolipid instillation) with either an isotype control mAb (LTF2, 500 μg/mouse) or an anti-CD1d mAb (19G11, 500 μg/mouse). Means ± SEM are shown (*n* = 3/group). α-GalCer, α-galactosylceramide; IFNγ, interferon gamma; *i*NKT, invariant natural killer T.
(TIF)

S11 Fig General synthesis of GD3. (TIF)

S12 Fig General synthesis of GM3. (TIF)

S13 Fig Stereo-controlled synthesis of GD3 (C24:1). (TIF)

S14 Fig Stereo-controlled synthesis of GM3 (C24:1). (TIF)

S15 Fig Synthesis of β-GlcCer. β-GlcCer, β-glucosylceramide.
(TIF)

S16 Fig Comparison of ^1^H NMR spectra of GD3 (C24:1, stereo-controlled versus general). ^1^H NMR, proton nuclear magnetic resonance.
(TIF)

S17 Fig Comparison of ^1^H NMR spectra of GM3 (C24:1, stereo-controlled versus general). ^1^H NMR, proton nuclear magnetic resonance.
(TIF)

S18 Fig HPLC of stereo-controlled GD3 (C24:1). HPLC, high-pressure liquid chromatography.
(TIF)

S19 Fig LC-MS of stereo-controlled GD3 (C24:1). LC-MS, liquid chromatography-mass spectrometry.
(TIF)

S20 Fig HPLC of stereo-controlled GM3 (C24:1). HPLC, high-pressure liquid chromatography.
(TIF)

S21 Fig LC-MS of stereo-controlled GM3 (C24:1). LC-MS, liquid chromatography-mass spectrometry.
(TIF)

S1 Table *N*-acyl chain analysis of DC-derived versus buttermilk-derived GM3. The acyl chain composition of GM3 from mouse unstimulated DCs or commercial bovine buttermilk was measured and compared by ES(-)-LC-MS/MS. Two sets of samples were measured and indicated as mean values. DC, dendritic cell; ES(-)-LC-MS/MS, electrospray(-)-liquid chromatography-mass spectrometry/mass spectrometry.
(XLSX)

S2 Table Primers used in this study. (XLSX)

S1 Data Numerical data used in this study. Numeric data shown in separate excel spreadsheets.
(XLSX)

## Abbreviations

^1^H NMR: proton nuclear magnetic resonance
α-GalCer: α-galactosylceramide
γδT: gamma delta T
Ag: antigen
β-GlcCer: β-glucosylceramide
CerS: ceramide synthase
CpG: cytosine-phosphate-guanine
DC: dendritic cell
ES(-)-LC-MS/MS: electrospray(-)-liquid chromatography-mass spectrometry/mass spectrometry
GSL: glycosphingolipid
HILIC-MS^2^: hydrophilic interaction liquid chromatography-tandem mass spectrometry
IFNγ: interferon gamma
iGb3: isoglobotrihexosylceramide
IL-2: interleukin 2
*i*NKT: invariant natural killer T
MHC: major histocompatibility complex
NKT: type I natural killer T cell
NP-HPLC: normal-phase high-pressure liquid chromatography
ODN: oligodinucleotide
TCR: T-cell receptor
Th1: T-helper 1
TLR: Toll-like receptor
WT: wild-type
