# Supplementary figures and images for: TLR9-mediated dendritic cell activation uncovers mammalian ganglioside species with specific ceramide backbones that activate invariant natural killer T cells
Source: PLoS Biol. 2019 Mar 1;17(3):e3000169. doi: 10.1371/journal.pbio.3000169 (PMC6420026; doi:10.1371/journal.pbio.3000169)

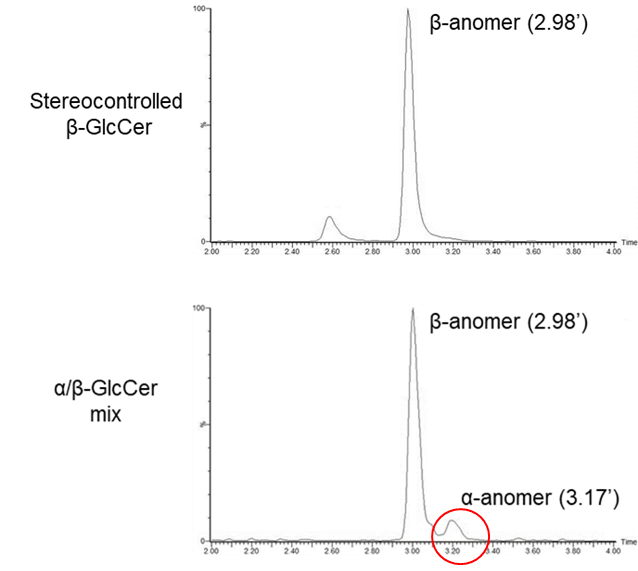

Supplement: S2 Fig — HILIC-MS2 of stereo-controlled β-GlcCer and an α/β-GlcCer mixture. α/β-GlcCer, α/β-glucosylceramide; HILIC-MS2, hydrophilic interaction liquid chromatography-tandem mass spectrometry. (TIF) [file pbio.3000169.s002.tif]
